# Ecosystem health appears neglected in the management of the human-macaque interface: A systematic review

**DOI:** 10.1016/j.onehlt.2024.100875

**Published:** 2024-08-20

**Authors:** Sukuman Rittem, Tithipong Plangsangmas, Simon R. Ruegg

**Affiliations:** a Epidemiology and Biostatistics, Life Science Zürich Graduate School, University of Zurich, Zurich, Switzerland; b Chulabhorn Royal Academy, Bangkok, Thailand; c Vetsuisse Faculty, Section of Epidemiology, University of Zurich, Zurich, Switzerland

**Keywords:** Human, Macaques, Transdisciplinary, Sustainability management, One health

## Abstract

Macaques (*Macaca* spp.) are reported in human-wildlife interaction in anthropogenic areas. The management of human-macaque interactions (HMI) requires an understanding of various perspectives and knowledge. One Health (OH) is a transdisciplinary approach to address the well-being and health of animals, humans, and ecosystems, which supports sustainable management through its three pillars: economy, ecology, and society. Thus, the OH approach could be applied to HMI management. To explore the HMI management within the context of the OH approach, we examined articles related to the management of HMI from 2013 to 2022 following the systematic review by PRISMA guidelines. Ninety-four publications were included in the study. Then, we extracted information on HMI framing, management activities, species, and location and categorized HMI framings and management activities into themes of three OH domains and three pillars of sustainability. We noticed an underrepresentation of the society and economy pillars in HMI management and the ecosystem health domain was the least explored in both the HMI and management activities. When we connected publications addressing all three pillars with OH domains in management activities, the number focused on ecosystem health (3/13) remained limited. The most frequently reported HMI theme was “crop feeding”(n=42) and management activities were “HMI management” (n=42). Most publications lacked any form of evaluation of the HMI management. The challenges to better consider ecosystem health in the HMI and to promote participatory governance present an opportunity to apply the OH approach in wildlife conservation and management.

## 1 Introduction

Human-wildlife interaction (HWI) encompasses both negative and positive interactions between humans and wildlife [1]. *Macaca* spp. have been involved in HWI due to their ability to easily adapt social life and natural behavior to anthropogenic environments. For example, they exhibit reduced grooming frequency and diversity as they spend mor time monitoring human activities [2]. Eco-tourism areas serve as locations for human-macaque interaction (HMI). It can be an example of the advantages for humans associated with having wildlife and potentially contribute to zoonotic disease transmitted between humans and animals [[3], [4], [5]]. People's perspectives toward macaques can vary being both positive and negative, depending on the different perceptions, cultural or social contexts [[6], [7], [8]]. The traditional esteem and religion relation for macaques motivates feeding and consequent unnatural population growth and macaque behavior change, as well as engagement for their protection [7,9]. In agricultural areas, people who had property destroyed by macaques estimated the severity of the problem in correlation to the value of the damage sustained. This is an example of negative HMI that leading to the human-macaque conflict (HMC) [7,[10], [11], [12]]. The interaction between humans and macaques has the potential to increase the risk of zoonotic disease transmission to humans [13]. It illustrates that the HMI covers various dimensions, including macaque behavior, ecology, economics, zoonotic diseases, religion, cultural beliefs, and societal aspects. Addressing these complex issues requires a systemic approach, relying on multiple perspectives for effective governance [14].

The One Health (OH) approach aims to mobilize multiple sectors, disciplines, and communities across different levels of society to integrate knowledge and collaborate for improved well-being in the three domains including humans, animals, and ecosystems. It posits that maintaining global health should involve a careful balance of well-being across the three health domains [15,16]. The impacts expected from a OH approach can be observed in three pillars of sustainability (economic, ecological and social pillars) to mitigate complex problems and maintain sustainable solutions [17,18]. The pillars of sustainability concept has been proposed to underpin project management in diverse areas such as, urban planning, climate change or sustainable agriculture [[19], [20], [21]]. Thus, the OH approach is a management option to apply to the HMI. Effective management in this context demand a comprehensive consideration of diverse factors, including societal, economic, and health aspects of both human and macaque populations, as well as the broader environmental context [12,22,23]. Therefore, we aimed to examine the application of the OH approach in the context of HMI. Our study investigated the research on HMI and its management, specifically related to concept of OH domains and pillars of sustainability, through the systematic review process. We included publications from 2013 to 2022, focusing on how they framed HMI and its management. Then, we assessed the HMI and management activities concerning human, animal and ecosystem health, as well as evaluated the relationship between HMI management activities and the three pillars of sustainability (society, economy, ecology).

## 2 Methods

### 2.1 Data collection

**Fig. 1:**
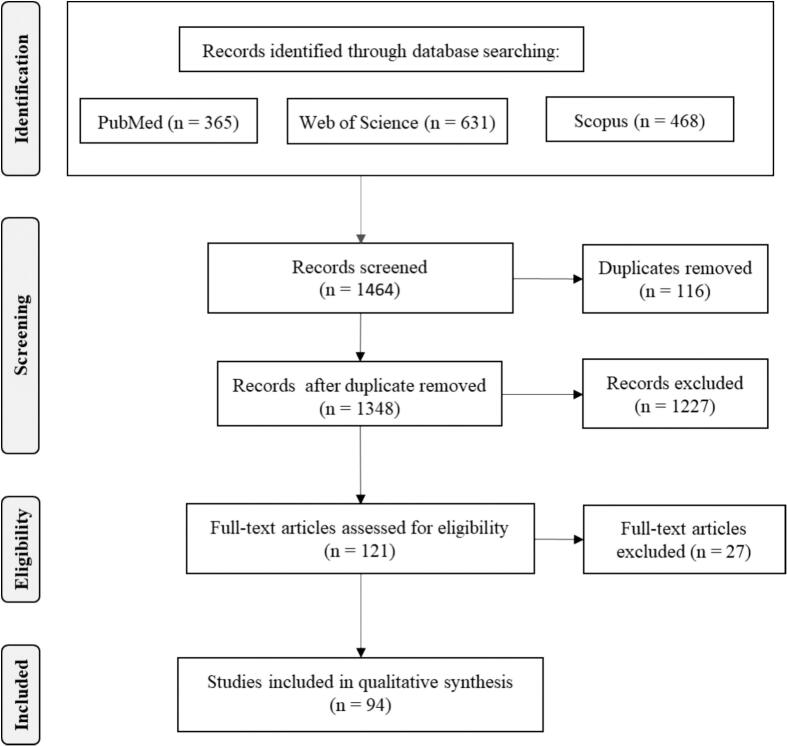
Flow chart according to the PRISMA guidlines showing the work flow with the number of publications included and exculded each step.

We conducted a qualitative systematic review according to the Preferred Reporting Items for Systematic Reviews and Meta-analysis (PRISMA) guidelines [24]. Each step was completed independently by two reviewers and subsequently discussed in case of disagreement. An initial literature search collected all original research articles and case reports from three databases including PubMed, Scopus, and Web of Science between 01/01/2013 to 31/12/2022. The following keywords were combined with Boolean operators:(“macaque” OR “Macaca” OR “human-monkey” OR “human-macaque”) AND (“mitigation” OR “resolve” OR “management” OR “intervention” OR “implementation” OR “strategy”) AND (“conflict” OR “interaction” OR “interface” OR “coexistence”). In the next step, we systematically screened full articles after completing the initial screening of titles and abstracts (Fig. 1). The exclusion criteria during this screening step comprised: 1) articles not published in English or lacking accessible full text; 2) articles not presented as comprehensive original research papers or case reports; 3) articles either lacking information on HMI management or did not provide a detailed description of HMI management; 4) articles unrelated to HMI in interface areas. Subsequently, data extraction was performed on the included articles.

### 2.2 Data analysis

#### 2.2.1 Framing development of HMI themes and HMI management activities

The following information was extracted from each publication: the year of publication, species of macaques, location of the study. Two reviewers of the author team independently classified the theme of the HMI and the management activities in a process based on grounded theory which is a qualitative method of analysis [25]. The coding of relevant keywords related to HMI and HMI management activities in the publications was conducted to develop initial themes. Subsequent articles with similar keywords or context were assigned to existing themes, with additional descriptors added as necessary. If none of the preceding themes matched the keywords or descriptions in the articles, a new theme was coined with relevant descriptors. When specific explanations of activities or purposes were provided, they were categorized into new specific groups. The themes were progressively adapted and complemented to incorporate the HMI and management activities effectively covered by the studies until all articles were thematically described. In the next step, the two reviewers discussed each publication together to reach a consensus on the allocation of the publication to the themes and definitions of each theme. We also extracted data related to the evaluation of the HMI management activities. The assessment was distinguished in three types of assessment [26]: 1) economic assessments including the assessment of time, budget and manpower related to the effectiveness of management activities, 2) impact assessments including macaque health and behavior, macaque population and human perspective assessment, and 3) the process evaluation of HMI management activities including the frequency of management activities.

#### 2.2.2 Identifying OH domains and pillars of sustainability in HMI

The HMI and HMI management activities were attributed to three domains of OH (human, animal and ecosystem health) [16]. The conceptualization of ecosystem health was established on the premise that it represents the sustained structural and functional integrity of ecosystems across time [27,28]. The definition of human health was adopted from the World Health Organization as a condition of holistic well-being encompassing physical, mental, and social dimensions [29]. In the case of animal health, we adapted the definition of health related to disease and welfare in both individual and population level [30,31]. The management activities were also attributed to the pillars of sustainability (society, economy, ecology dimensions) based on the concept by Purvis et al. [17]. A detailed description of the OH domains and pillars of sustainability can be found in Table 1. The classification of HMI and management activities within the domains of OH and the pillars of sustainability was independent of their thematic framing. Each HMI and management activity could be categorized into multiple OH domains or sustainability pillars. Consequently, each article could be assigned to different OH domains or pillars, with each attribution counted separately. The number of OH domains and pillars related to HMI and HMI management activities within the publications was investigated to assess the coverage of the OH approach. We then used a chi-squared test in R (version 4.3.0) to compare the number of HMI and management activities associated to the OH domains.

**Table 1:** Criteria used to attribute HMI and management activities to the pillars of sustainability and the domains of OH.

Pillars of sustainability		Domains of OH	
Pillar	Definition	Domain	Definition
Ecology	Management activities focus on the ecosystem and factors affecting the ecosystem such as spatial distribution in both human and animal, habitat modification, live matters, inanimate objects, and pathogens	Ecosystem health	Interactions and activities concern physical, chemical, and biological factors external to humans and animals which relate to the balancing and maintaining ecosystem.
Society	Management activities focus on human social system and their livelihoods such as measures affecting public education, public engagement, beliefs, politics, or government policies that can support human well-being	Human health	Interactions, activities and management of environment concern the impact on human well-being and function including aspects of disease, physiological function, mental stability, and social development.
Economy	Management activities focus on resource allocation and management such as income, compensation, employment, and resource utilization for sustainable well-being of human and environment	Animal health	Interactions and activities concern the impact on animal well-being including aspects of disease, animal life and welfare, natural behavior, animal social networks and population balancing.

## 3 Results

### 3.1 General observations

In total 1464 publications were identified of which 94 were retained for further analysis (Appendix S1). Most publications were reporting from studies in India (*n* = 22) followed by Malaysia [10] and Indonesia [10]. Fifteen macaque species were mentioned, and one publication did not identify the species. The detailed number of publications by country and macaque species are reported in Table 2.

**Table 2:** Coverage in publications between 2013 and 2022, by country of study and macaque species.

Country	No. of publications	Species	No. of publications
Algeria	2	Barbary macaques (*Macaca sylvanus*)	2
Bangladesh	3	Rhesus macaques (*Macaca mulatta*)	3
China	8	Rhesus macaques (*Macaca mulatta*)	5
		Tibetan macaques (*Macaca thibetana*)	2
		*Macaca* spp.	1
Gibraltar	1	Barbary macaque (*Macaca sylvanus*)	1
India	22⁎	Arunachal macaque (*Macaca munzala*)	1
		Bonnet macaques (*Macaca radiata*)	8
		Rhesus macaques (*Macaca mulatta*)	14
		Lion-tailed macaques (*Macaca silenus*)	2
		Long-tailed macaques (*Macaca fascicularis*)	1
Indonesia	10⁎	Booted macaques (*Macaca ochreata*)	2
		Long-tailed macaques (*Macaca fascicularis*)	5
		Moor macaques (*Macaca maura*)	3
		Tonkean macaques (*Macaca tonkeana*)	2
		Heck's macaques (*Macaca hecki*)	1
Japan	9	Japanese macaques (*Macaca fuscata*)	9
Malaysia	10⁎	Long-tailed macaques (*Macaca fascicularis*)	9
		Southern pig-tailed macaques (*Macaca nemestrina*)	4
Morocco	5	Barbary macaques (*Macaca sylvanus*)	5
Nepal	7	Assamese macaques (*Macaca assamensis*)	2
		Rhesus macaques (*Macaca mulatta*)	5
Philippines	1	Long-tailed macaques (*Macaca fascicularis*)	1
Singapore	4	Long-tailed macaques (*Macaca fascicularis*)	4
Sri Lanka	6	Toque macaque (*Macaca sinica*)	6
Thailand	6	Long-tailed macaques (*Macaca fascicularis*)	5
		Rhesus monkeys (*Macaca mulatta*)	1
USA	2	Rhesus monkeys (*Macaca mulatta*)	2

⁎ The total number of publications in the country is lower than the total number of publications in the species because some publications mentioned more than one species.

### 3.2 Themes of HMI and HMI management activities

Using the grounded theory approach, HMIs were classified into 10 themes (Table 3) and the number of publications for each theme is presented in Table 4. Similarly, the management activities at the human-macaque interface were categorized into 11 themes (Table 5). The number of publications that were counted for each theme is presented in Table 4.

**Table 3:** Themes of HMI and description extracted from the publications.

Theme of HMI	Description
Crop feeding	Activities such as foraging or entering agricultural plantations by macaques resulting in the destruction of plantations.
Macaque access human properties	Macaques enter both indoor and outdoor areas of households, engaging in activities that result in damage to real estate, vehicles, and privately owned plants, excluding crops.
Actions of macaques toward humans	Activities of macaques directed at humans or domestic animals, such as aggressive behavior, attacking or snatching, which cause human dissatisfaction or damage.
Encroachment of macaque habitat	Human activities on macaque habitat including deforestation, expansion of construction, or recreational activities.
Actions of humans and domestic animals toward macaques	Activities by humans and domestic animals directed at macaques, including abuse, violence or hunting for consumption.
Pet keeping	Humans keep macaques as a pet.
Disease transmission and health concern	Activities arise regarding infectious diseases transmitted among macaques, between macaques and humans, and between macaques and other species. Additionally, it included any topic that concern for the health status of both humans and macaques.
Overlapping land use	Activities involving encounters between humans and macaques occur due to competition or resource sharing, such as habitat or food, in overlapping areas.
Macaque behavior change	The alteration of macaque behavior from its natural way of living caused by HMI. These behaviors can be the cause of conflicts and affect humans or macaques. For example, macaques beg food from tourists or macaques change their activity budget or status of their groups.
Unidentified HMI theme	If the manuscript does not mention the specific HMI theme

**Table 4:** Coverage given to different themes of the HMI causing conflict and HMI management activities in publications between 2013 and 2022.

Theme of HMI	No. of publications	Theme of management activities	No. of publications
Crop feeding	42	HMI management	42
Actions from macaques toward humans	36	Biological data support for management activities	39
Macaque behavior change	33	Crop, properties, and livestock protection	33
Macaque access human properties	31	Macaque population management	30
Macaque habitat invasion	27	Education	29
Overlapping land use	23	Macaque habitat management	26
Actions from human and domestic animal toward macaques	22	Land use management	23
Disease transmission and health concern	21	Public engagement	19
Pet keeping	7	Park management	18
Unidentified HMI theme	1	Infectious disease management	14
		Compensation	12

Note: The overall sum of counts in the table is greater than the total number of papers (*n* = 94) reviewed because many studies report more than one cause of conflict or mitigation activity.

**Table 5:** Themes of HMI management activities and description extracted from publications.

Theme of HMI management activities	Description
Education	Activities related to knowledge transfer such as school interventions, training, knowledge sharing or information campaigns about the human-macaque-coexistence.
Public engagement	Activities aimed at facilitating the collaboration and information exchange between citizens, stimulate their concern about macaques or environmental conservation or respond their need requirement.
Macaque food and habitat management	Activities aimed to provide appropriate habitat and accessible food for macaque such as providing resource of food and protecting plants that support natural macaque behavior such as foraging and comfort behavior.
Land use management	Activities aimed to arrange the spatial distribution of land use to allow for peaceful coexistence, including urban and construction planning, or agricultural strategies such as changing crop species, choosing non-edible plantation for macaques, announce protected areas or managing buffer zone areas around protected areas.
Macaque population management	Activities targeted to control the macaque population such as birth control, relocation, translocation, or culling
Infectious disease management	Activities related to infectious diseases in both human and animal populations and diseases transmission between species, such as monitoring, surveillance, disease prevention or disease eradication, or biosafety measures.
Biological data support for HMI management	Activities that use information of biological data support management actions or policy. The example of biological data is macaque behavior data, macaque food and habitat data, ecological data, or genetic and diversity data
Human-macaque interaction management	Activities aim to reduce the cause of conflict from HMI in coexisting area such as prohibition on human activities by regulation and laws, improving areas to reduce HMI problems, facilitate the changing human habit or activities to reduce the agonistic interaction.
Park management	Activities target to mitigation in national park such as tourism management, employment, macaques feeding spot and method management focusing on the park, information and knowledge sign and portal management
Crop, property, and livestock protection	Actions by private properties owner aim to prevent and expel macaques such as guarding, fencing, making a loud noise, using firecracker or lighting, shooting, or poisoning
Compensation	Payments to provide a monetary equivalent for the experienced damage or funding aim to alleviate the conflict

The total number of publications that evaluated the effectiveness of management activities is thirty-six publications (38%). Among these publications, twenty-eight (28/36) did an impact analysis which include sixteen publications assessing people's perspective toward macaques in both positive and negative and twelve publications collecting biological data. Additionally, six (6/36) studies conducted a process evaluation, and two (2/36) performed an economic assessment. In eleven impact evaluations (11/28), there were quantitative findings discussing people's perspectives on the most effective mitigation activities. Among these articles, the top-rated management activity was macaque population control or translocation (5/11), followed by guarding (2/11), compensation (2/11), increased food resources for macaques (1/11), and waste management (1/11).

### 3.3 Distribution of the OH domains and pillars of sustainability

**Fig. 2:**
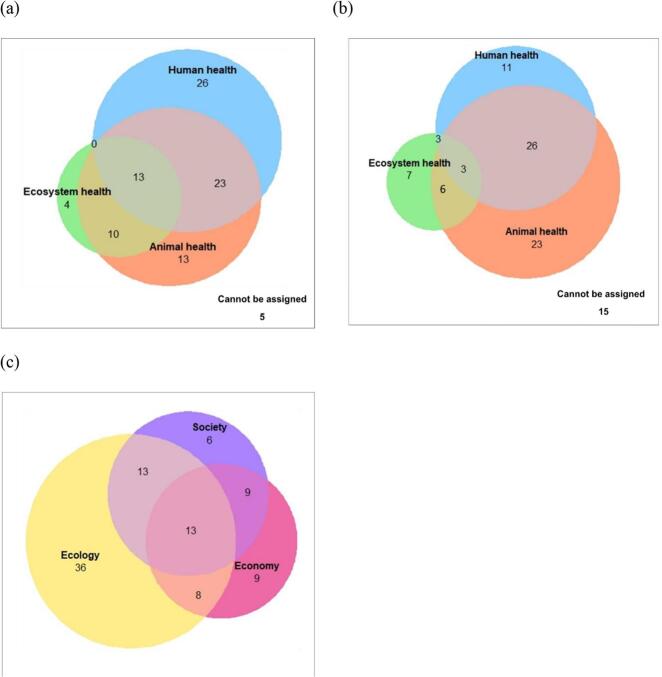
The attribution of publications (n = 94) to the OH domains (human, blue; animal orange, ecosystem green) of HMI (a), of HMI management (b), and to the pillars of sustainability (ecology yellow, society purple, economy pink) for HMI management (c). (For interpretation of the references to colour in this figure legend, the reader is referred to the web version of this article.)

The number of publications considering one or more OH domains is represented in Fig. 2. We identified eighty-nine publications that have the HMI theme related to the OH domain, while five publications could not be assigned (Fig. 2a.). For the HMI management activities, seventy-nine publications were linked to the OH domains, and fifteen publications could not be included (Fig. 2b). In 74 of 94 articles (79%), the OH domain in which the HMI management activities are implemented is different from the domain of the HMI framing, i.e. where impacts of these activities would be expected. And a vast majority of management activities (60/79, 76%) are implemented in the human or animal health domain.

There are significantly more HMI framings than management activities in all OH domains (df = 3, χ 2 = 8.75, *p* = 0.033). We found forty-three articles referring to HMI in one OH domain, thirty-three in two, and thirteen publications considering all three domains. HMI management activities were reported in only one domain by forty-one articles, thirty-five mentioned two domains, and three considered all three OH domains.

Ninety-four publications worked within the realm of pillars of sustainability. The number of publications considering one or more pillars is represented in Fig. 2C. There are fifty-one publications referring to one pillar, thirty publications referring to two pillars and thirteen publications were assigned to all three pillars.

## 4 Discussion

### 4.1 Consideration of ecosystem health in HMI

Our results revealed that out of 94 included publications only thirteen publications to which we can attribute HMI management activities to all pillars of sustainability and three mentioned all OH domains. Among the publications in which management activities occurred in all pillars (13/94), we found two of them also covered all OH domains. Seven publications included management activities in animal and human health, and one in ecosystem and human health. Two publications covered management activities only in the domain of animal health, and one publication mentioned management activities that could not be attributed to any OH domain due to incompatibility with our criteria. Therefore, publications that address all three pillars are limited, with the majority (10/13) failing to consider the domain of ecosystem health.

The findings regarding the OH domains exhibit a similar pattern. HMI framings predominantly give precedence to human health, while emphasizing the management of animal health. The rationale behind this result could be that some studies observed the human perspective, where participants focused on problems or impacts caused by macaques. In contrast, when addressing management issues, people usually concentrate on ways to manage the animals [[32], [33], [34]]. Comparing all OH domains, ecosystem health exhibited the smallest number in both HMI framings and management activities. Although the publications we identified focused on conservation efforts that involve education and sustainable program development, they did not directly address management strategies aimed at balancing and maintaining the ecosystem*.* This trend aligns with other issues beyond OH or HMI. Data from the Ecosystem Service Valuation Database, which represents the largest repository of studies on ecosystem service valuation, reported that 58% of publications did not mention ecosystem health [35]. The management of ecosystem health in ocean ecosystems, industrial activities, the utilization of natural energy, and ecotourism also received low attention [36]. As is valid for the whole of OH, effectively integrating socioeconomic, biophysical, biogeochemical, and public-policy dimensions is a challenge and a crucial factor in devising and maintaining effective strategies for ecosystem health [27,37].

Negative impacts on ecosystem health emerge when governance, policy and practice lacks consideration for maintaining healthy ecosystems, as seen in practices like deforestation or over hunting wild animals for crop protection [38]. The two publications that cover all OH domains and pillars of sustainability illustrate what a comprehensive approach could look like, even though they did not explicitly use the term OH. Mildra et al. [39] compare the human wildlife interface (including macaques) within and outside a protected area in India. Aspects of their comparison are state compensation in case of human death or injury, cattle predation, or crop damage. They also look at the implementation of physical barriers, crop protection through scaring wildlife to protect stock and crop. Then at reducing damage to human livelihoods by wildlife by enhancing wildlife conservation efforts such as protection and management capabilities, protecting wildlife habitat, maintaining sustainable populations, constructing percolated ponds, and relocating conflict animals. Finally, they cover endorsement of human livelihoods and social inclusion by supplying seeds, developing eco-tourism, supporting anti-poaching camps, and conducting public awareness programs, and implementing a law for community support and involvement. Lee & Davey [40] interviewed visitors to a country park in Hong Kong ascribed to social contact with wild rhesus macaques. The thematic analysis of the interviews comprises monkey health and well-being, the natural appearance of the plantations, and the mental health benefits visitors receive from human-macaque-interactions. Moreover, the visitors also considered the contribution of the park to society, to the economy through tourism, as well as to nature. Thus, the challenge to better consider ecosystem health in HMI framing presents an opportunity to apply the OH approach in wildlife conservation and sustainable management [41].

On the other hand, recommending more management activities targeted at ecosystem health may not necessarily add value, because due to the interconnectedness of the social-ecological system, HMI framing or management activities in one OH domain can have repercussions on the others, and addressing ecosystem health is inherently difficult [[42], [43], [44]]. This is suggested by the observation of Moroccan project, where enhancing human health and preventing rabies in dogs facilitates public participation, encourages people to engage in disease prevention, and supports macaque conservation efforts [45].

### 4.2 Effectiveness of HMI management activities

Macaque population control and translocation were the most effective mitigation activities, but the implementation varied across study sites. Some authors even argue that, despite being considered effective, macaque translocation did not lead to a sustainable resolution of the conflict [46,47]. While overall only a minority of studies included an evaluation, most (28/36) assessed the impact, but only two conducted an economic evaluation. The quantitative evidence for effectiveness we were able to collect is scarce. The predominance of assessing people's perceptions over collecting biological data leads us to assume that assessing the human perspective is more convenient, while biological data collection is more cumbersome. The latter is best illustrated by studies collecting specific indicators and using diverse approaches to assess e.g. the social status of macaques, better understand their behavior, land use, and monitor macaque population size [22,[48], [49], [50]]. Our assumption is further supported by the observation that most publications lack any form of evaluation of the HMI management. Such evaluations require a mixed methods approach including qualitative and quantitative data. The evaluation framework in questionnaire development to measure the effect of intervention program for human-wildlife conflict mitigation such as the investigation of The New York NeighBEARhood Watch Program [51]. Other method to evaluate the human-wildlife interaction management such as, the observation of human behaviors and action change, ecological movement or monitoring strategies [52,53]. For OH and other integrated approaches to health, a number of dedicated evaluation tools are available and could adapted to management activities of HMI. For example, an OH evaluation framework was developed considering outcomes in society, economy and ecology, as well as providing a tool to assess the integration of knowledge including citizen participation throughout the process [54].

### 4.3 Society and economy pillars are underrepresented

From a sustainability perspective, the HMI management activities are mostly implemented in the ecology pillar, followed by economy and society. The less predominant given to the society pillar aligns with the trend observed in the review of the Sustainable Development Goals between 2010 and 2015 and in two sustainability projects by the Swedish Environmental Protection [55,56]. In these reviews, different professions emphasized different pillars: e.g. environmentalists prioritized the ecology in rural areas, focusing on wildlife and nature preservation, whereas policymakers tended to prioritize the economy. According to Giddings et al. [57], this difference arises from the perception of the environment as separate from humans. Consequently, sustainable resource management can lead to conflicts of interest among stakeholders, involving aspects like land development versus habitat conservation, as well as the balance between economic necessities and park functioning [44]. However, like the intricate connections between the OH domains, overlooking certain pillars or employing sectoral approaches may have adverse effects on the whole social-ecological system. For instance, crop protection techniques often focus on economic considerations, neglecting the societal impacts that may lead to children not attending school as they are occupied with guarding crops, or if the ecology pillar took precedence, for example restricting the human use in overlapping areas, or lacking compensation for HMI problems led to citizens harboring negative attitudes toward macaques [32,38]. This resulted in reduced participation and increased resistance against conservation efforts [22]. Considering coverage dimensions is challenging and requires careful assessment. Projects engaging with stakeholders and incorporating more perspectives from residents tended to prioritize economy and society. These included supporting ecotourism, addressing crop losses from primate foraging, and addressing issues in co-habitat areas. On the downside, these areas tended to lack ecological considerations [58].

### 4.4 The themes of HMI and management activities

The most prevalent theme to frame HMI was ‘crop feeding’. Crops were identified as the most attractive food resource for macaques compared to e.g., kitchen supplies, tree fruits, garbage, or natural food [7]. In Nepal, macaques were reported the most important species involved in crop feeding [59]. The most influential factors for the frequency of crop feeding were the different crop types, their distance to macaque habitat, and crop protection measures [34]. The second most common theme was ‘actions of macaques toward humans’. The activities of macaques that were considered a nuisance included natural and unnatural behavior directed at humans or domestic animals [49,60]. The reasons for such behavior could be food competition, territorial or individual defense, and can thus not be entirely dissociated from human activity [9,49,61]. Meanwhile, the reciprocal behavior of ‘actions of humans and domestic animals toward macaques’ was thought to result from discontent with macaque behavior, or defensive action [49,62], but was only considered in seventh place as an issue at the human-macaque interface. The third most important framing of the HMI was ‘macaque behavior change’, which could also be an origin of other HMI causing conflict such as ‘macaque access human properties’, ‘action of macaques toward humans’ or ‘disease transmission and health concern’ [[63], [64], [65]]. Among the territorial themes ‘overlapping land use’ was often driven by the loss of natural macaque habitat and expanding human habitat. In these areas, wild animals primarily utilize resources for survival, while humans exhibit more complex motivations [66,67]. Eventually, the perspective of locals in the interface area toward macaques was determined by their economic loss [68]. The more unilateral ‘encroachment of macaque habitats’ was driven by more political or systemic factors, such as deforestation, mining, expanding crop plantations, fishing in overlapping areas, and policies related to land use development [69,70].

One of the least concerns was ‘disease transmission and health concerns’. Among these, surveillance, diagnosis, and transmission prevention of zoonotic diseases from macaques to humans appeared most prominent. Seventeen of 21 publications discussed zoonotic diseases, two publications referred to both zoonotic and anthroponotic diseases, one publication addressed human health, and another one focused on animal health. Regarding the risk for humans, particularly intestinal parasites, bacteria, viruses, and *Plasmodium* spp. were mentioned [5,71,72]. The risk for macaques was identified in association with feeding practices, namely the transmission of intestinal parasites and bacteria, as well as *Cryptosporidium hominis* [[73], [74], [75]]. Macaques that consumed provisioned food exhibited higher microbial richness compared to the counterparts in the wild [76,77]. But disease transmission could also be a consequence of other HMI such as ‘overlapping land use’, mutual habitat invasion, direct interactions, or keeping pet macaques [68,75,78].

In terms of HMI management activities, the most frequent theme was ‘HMI management’. Together with ‘park management’ it comprises activities to diminish human activities considered to cause conflicts at the human-macaque interface but focuses on activities that reduce conflict opportunities outside spaces dedicated to wildlife. Activities include improving garbage management and modified housing to prevent damage from macaques [79]. Some authors suggested that integrated management strategies should additionally include natural sources of food and water, macaque population control, and preventing macaques from accessing human food [8,80]. Eco-tourism was suggested to balance the societal, economic, and ecological value, but there are also some reservations regarding the consequences of such interactions [58,81]. The concerns are effects on the natural behavior of macaques, their activity budget and risks of disease transmission [82]. Consequently, eco-tourism with direct HMIs was only recommended under the precondition of appropriate management, and staff and visitor education focusing on animal behavior, welfare, and health [81,83]. The second most common management theme was ‘biological data support for activities management’. The data has been used to understand feeding behavior and nutrition of macaques. It was considered very important in solving the problem of crop feeding [84,85]. The management activities also included camera traps, telemetry data, mobile phone reporting and mathematical modelling to monitor macaque behavior and develop adaptive management plans [50,86,87]. The third management theme comprised ‘crop, property and livestock protection’. The protection activities were mentioned in different ways, e.g. with offensive measures such as guarding or expelling macaques by throwing stones, firecrackers, dog guarding or by passive means such as using barriers [88,89]. Human guarding was frequently mentioned as a means to manage the HMI, but it results in significant time loss for farmers or produces substantial social impact such as guarding children were not able to attend school [46,90,91].

For long term peaceful cohabitation, it was especially recommended to increase public engagement and education [7,23]. Type of knowledge in conservation education was observed the different outcome for learners [92]. However, there are some questions about increasing conservation knowledge and changing long-term behavior and attitude toward the conservation of people [93]. Interestingly, the top-down approach through education [29] seems more popular than public participation [19], which is more cumbersome and requires expertise and long-term commitment. This may indicate a further reason to deploy interdisciplinary and transdisciplinary for the management of the HMI. Among setting-based approaches were the themes ‘macaques habitat management’ and ‘land use management’. The former was distinguished from the latter because it focuses on environmental improvement for macaques to reduce their motivation to leave their habitat in search for anthropogenic food sources [79]. ‘Land use management’ included the spatial distribution of crops and constructions, declaration of protected areas or buffer zones or the establishment of areas for co-habitation to reduce attractivity for macaques, improve the context for HMI and prevent conflicts [34,68]. Changing crop species, improving crop protection strategies and trade-offs between urbanization and ecosystem conservation were reported as key strategies and commonly used together to alleviate the crop feeding problems [70,91,94]. Corresponding to the little concerns about disease transmission, ‘infectious disease management’ was not a common intervention. The theme comprised disease monitoring to better understand the ecology, biology, and behavior of hosts and infective agents, with the aim of effectively controlling zoonotic diseases. The choice of appropriate diagnostic tools is considered important to balance public health and conservation concerns [65,71]. Also, elements of HMI management and education were mentioned to reduce the opportunity of zoonotic spread in eco-tourism and co-inhabited areas [63,95].

## 5 Conclusion

Considering the coverage dimensions in the HMI and evaluation of implementation is the reason to adopt the OH approach that encourages interdisciplinary and transdisciplinary working in our study. Adopting the concept of OH domains and pillars of sustainability to HMI and their management activities was challenging, as the definitions and contexts in the articles did not explicitly address the relation to OH domains or sustainability pillars in their objectives or outcomes. To reduce personal bias and misleading evaluations, duplicate data extraction was conducted independently by two review authors. Nevertheless, this analysis, based on grounded theory, may not fully correspond to conclusions drawn by other reviewers. Additionally, the exclusion of publications due to language restrictions and limited access during the screening step is a limitation, potentially introducing bias to the included data. The study focuses on manuscripts explicitly elaborating on the management of HMI; therefore, conflicts caused by HMI may not be fully covered in our review.

Our finding demonstrated that HMI and HMI management predominantly related to human health, while emphasizing the management of animal health. Publications that address all three pillars of sustainability, including ecosystem health, were the fewest, even though the realm of ecology pillar was the most frequent in HMI management. Crop feeding was the most frequent in HMI, whereas crop protection, directly responding to this activity, was not the most prominent in HMI management. It could imply that HMI management activities with boarder dimensions could mitigate various problems. Developing a framework to evaluate the effectiveness of the HMI management remains a challenge for further research.

## CRediT authorship contribution statement

**Sukuman Rittem:** Conceptualization, Data curation, Formal analysis, Methodology, Project administration, Writing – original draft, Writing – review & editing. **Tithipong Plangsangmas:** Conceptualization, Data curation, Formal analysis, Methodology. **Simon R. Ruegg:** Conceptualization, Methodology, Supervision, Writing – original draft, Writing – review & editing.

## Declaration of competing interest

The authors declare that they have no known competing financial interests or personal relationships that could have appeared to influence the work reported in this paper.

The author is an Editorial Board Member/Editor-in-Chief/Associate Editor/Guest Editor for *[Journal name]* and was not involved in the editorial review or the decision to publish this article.

## Data Availability

Data will be made available on request.

## Appendix A Supplementary data

Supplementary material 1 Image 1

Supplementary material 2 Image 2
