# Supplementary material for: Ecosystem health appears neglected in the management of the human-macaque interface: A systematic review
Source: One Health. 2024 Aug 20;19:100875. doi: 10.1016/j.onehlt.2024.100875 (PMC11381846; doi:10.1016/j.onehlt.2024.100875)
Supplement: Supplementary file 1 — Supplementary material 1 [file mmc1.docx]

**Supplement 1.** Reference list of all studies included in the systematic review.

Abdul-Latiff, M. A. B., Abdul-Patah, P., Yaakop, S., & Md-Zain, B. M. (2017). Aiding pest control management of long-tailed macaques (*Macaca fascicularis* *fascicularis*) in Malaysia by using molecular markers of mitochondrial DNA. *AIP Conference Proceedings*, *1891*(October), 020003. https://doi.org/10.1063/1.5005336

Abudul-Nasir, N. S., Osman, N. A., Hashim, Z. F., Baharudin, Z., Abdullah, M. I., Isa, Z., & Md-zain, B. M. (2021). Assessing perceptions and solutions to human-long tailed macaques (*Macaca fascicularis*) conflict in the Universiti Kebangsaan Malaysia campus, Bangi, Selangor, Malaysia. *Malayan Nature Journal*, *73*(2), 187–197.

Alami, A. El, & Chai, A. (2014). Distribution of the endangered Barbary macaque and human‐macaque interaction in the tourist region of Ouzoud, Central High Atlas of Morocco. *African Journal of Ecology*, *53*, 375–377.

Amir, A., Shahari, S., Liew, J. W. K., de Silva, J. R., Khan, M. B., Lai, M. Y., Snounou, G., Abdullah, M. L., Gani, M., Rovie-Ryan, J. J., & Lau, Y. L. (2020). Natural Plasmodium infection in wild macaques of three states in peninsular Malaysia. *Acta Tropica*, *211*(June), 1–6. https://doi.org/10.1016/j.actatropica.2020.105596

Anand, S., Binoy, V. V., & Radhakrishna, S. (2018). The monkey is not always a God: Attitudinal differences toward crop-raiding macaques and why it matters for conflict mitigation. *Ambio*, *47*(6), 711–720. https://doi.org/10.1007/s13280-017-1008-5

Anand, S., & Radhakrishna, S. (2020). Is human–rhesus macaque (*Macaca mulatta*) conflict in India a case of human–human conflict? *Ambio*, *49*(10), 1685–1696. https://doi.org/10.1007/s13280-020-01324-w

Anand, S., Vaidyanathan, S., & Radhakrishna, S. (2021). The role of landscape structure in primate crop feeding: Insights from rhesus macaques (*Macaca mulatta*) in Northern India. *International Journal of Primatology*, *42*(5), 764–780. https://doi.org/10.1007/s10764-021-00238-y

Anderson, C. J., Van De Kerk, M., Pine, W. E., Hostetler, M. E., Heard, D. J., & Johnson, S. A. (2019). Population estimate and management options for introduced rhesus macaques. *The Journal of Wildlife Management*, *83*(2), 295–303. https://doi.org/10.1002/jwmg.21588

Balasubramaniam, K. N., Kaburu, S. S. K., Marty, P. R., Beisner, B. A., Bliss-Moreau, E., Arlet, M. E., Ruppert, N., Ismail, A., Anuar Mohd Sah, S., Mohan, L., Rattan, S., Kodandaramaiah, U., & McCowan, B. (2021). Implementing social network analysis to understand the socioecology of wildlife co-occurrence and joint interactions with humans in anthropogenic environments. *Journal of Animal Ecology*, *90*(12), 2819–2833. https://doi.org/10.1111/1365-2656.13584

Balasubramaniam, K. N., Marty, P. R., Arlet, M. E., Beisner, B. A., Kaburu, S. S. K., Bliss-Moreau, E., Kodandaramaiah, U., & McCowan, B. (2020). Impact of anthropogenic factors on affiliative behaviors among bonnet macaques. *American Journal of Physical Anthropology*, *171*(4), 704–717. https://doi.org/10.1002/ajpa.24013

Baral, K., Sharma, H. P., Kunwar, R., Morley, C., Aryal, A., Rimal, B., & Ji, W. (2021). Human wildlife conflict and impacts on livelihood: A study in community forestry system in mid-hills of Nepal. *Sustainability (Switzerland)*, *13*(23). https://doi.org/10.3390/su132313170

Bashyal, A., Sharma, S., Koirala, N., Shrestha, N., Aryal, N., Yadav, B. P., & Shrestha, S. (2022). Crop raiding and livestock predation by wildlife in Khaptad National Park, Nepal. *Journal of Threatened Taxa*, *14*(11), 22156–22163.

Beisner, B. A., Balasubramaniam, K. N., Fernandez, K., Heagerty, A., Seil, S. K., Atwill, E. R., Gupta, B. K., Tyagi, P. C., Chauhan, N. P. S., Bonal, B. S., Sinha, P. R., & McCowan, B. (2016). Prevalence of enteric bacterial parasites with respect to anthropogenic factors among commensal rhesus macaques in Dehradun, India. *Primates*, *57*(4), 459–469. https://doi.org/10.1007/s10329-016-0534-2

Beisner, B. A., Heagerty, A., Seil, S. K., Balasubramaniam, K. N., Atwill, E. R., Gupta, B. K., Tyagi, P. C., Chauhan, N. P. S., Bonal, B. S., Sinha, P. R., & McCowan, B. (2015). Human-wildlife conflict: Proximate predictors of aggression between humans and rhesus macaques in India. *American Journal of Physical Anthropology*, *156*(2), 286–294. https://doi.org/10.1002/ajpa.22649

Bhandari, A., Joshi, R., Thapa, M. S., Sharma, R. P., & Rauniyar, S. K. (2022). Land cover change and its impact in crop yield: A case study from Western Nepal. *Scientific World Journal*, *2022*, 1–9. https://doi.org/10.1155/2022/5129423

Boonkusol, D., Sanyathitiseree, P., Thongyuan, S., & Jangsuwan, N. (2018). Population and behavior surveys of long-tailed macaque (*Macaca fascicularis*) in the old town, Lopburi province. *OnLine Journal of Biological Sciences*, *18*(2), 226–236. https://doi.org/10.3844/ojbsci.2018.226.236

Boumenir, M., Hornick, J. L., Taminiau, B., Daube, G., Brotcorne, F., Iguer-Ouada, M., & Moula, N. (2022). First descriptive analysis of the faecal microbiota of wild and anthropized barbary macaques (*Macaca sylvanus*) in the region of Bejaia, Northeast Algeria. *Biology*, *11*(2). https://doi.org/10.3390/biology11020187

Cabral, S. J., Prasad, T., Deeyagoda, T. P., Weerakkody, S. N., Nadarajah, A., & Rudran, R. (2018). Investigating Sri Lanka’s human-monkey conflict and developing a strategy to mitigate the problem. *Journal of Threatened Taxa*, *10*(3), 11391–11398. https://doi.org/10.11609/JOTT.3657.10.3.11391-11398

Carne, C., Semple, S., MacLarnon, A., Majolo, B., & Maréchal, L. (2017). Implications of tourist–macaque interactions for disease transmission. *EcoHealth*, *14*(4), 704–717. https://doi.org/10.1007/s10393-017-1284-3

Chen, M., Tan, A. D. J., Quek, W. L., & Chahed, H. (2022). A proposal for a technology-assisted approach to wildlife management in Singapore. *Pacific Conservation Biology*, *29*, 1–16. https://doi.org/10.1071/PC21055

Chijiiwa, A., & Nishimura, A. (2021). Effects of vegetation structure on the habitat utilization of free-ranging Japanese macaques (*Macaca fuscata*) in a coniferous plantation area. *Mammal Study*, *46*(2), 143–152. https://doi.org/10.3106/ms2020-0069

Cui, Q., Ren, Y., & Xu, H. (2021). The escalating effects of wildlife tourism on human–wildlife conflict. *Animals*, *11*(5), 1–16. https://doi.org/10.3390/ani11051378

Dittus, W. P. J. J., Gunathilake, S., & Felder, M. (2019). Assessing public perceptions and solutions to human-monkey conflict from 50 years in Sri Lanka. *Folia Primatologica*, *90*(2), 89–108. https://doi.org/10.1159/000496025

Dzulhelmi, M. N., Suriyanti, S., & Manickam, S. (2019). Population, behaviour and conservation status of long-tailed macaque, *Macaca fascicularis* and southern pig-tailed macaque, *Macaca nemestrina* in paya bakau park, perak, Malaysia. *Journal of Animal and Plant Sciences*, *29*(2), 611–618.

Enari, H. S., & Enari, H. (2021). Reasons for the dissatisfaction of residents with the recent reduction in the amount of crop damage by Japanese macaques-a case study in a typical depopulating prefecture in northern Japan. *Mammal Study*, *46*(2), 153–160. https://doi.org/10.3106/ms2020-0060

Enari, H., & Sakamaki–Enari, H. (2013). Resource use of Japanese macaques in heavy snowfall areas: Implications for habitat management. *Primates*, *54*(3), 259–269. https://doi.org/10.1007/s10329-013-0343-9

Fernando, S. U., Udagama, P. V., & Fernando, S. P. (2022). Effect of urbanization on zoonotic gastrointestinal parasite prevalence in endemic toque macaque (*Macaca sinica*) from different climatic zones in Sri Lanka. *International Journal for Parasitology: Parasites and Wildlife*, *17*(December 2021), 100–109. https://doi.org/10.1016/j.ijppaw.2021.12.007

Gamalo, L. E., Baril, J., Dimalibot, J., Asis, A., Anas, B., Puna, N., & Paller, V. G. (2019). Nuisance behaviors of macaques in Puerto Princesa Subterranean River National Park, Palawan, Philippines. *Journal of Threatened Taxa*, *11*(3), 13287–13294. https://doi.org/10.11609/jott.4702.11.3.13287-13294

Gangadharan, A., Vaidyanathan, S., & St. Clair, C. C. (2016). Categorizing species by niche characteristics can clarify conservation planning in rapidly-developing landscapes. *Animal Conservation*, *19*(5), 451–461. https://doi.org/10.1111/acv.12262

Ganguly, I., & Chauhan, N. S. (2019). How perception of local people towards rhesus macaque (*Macaca mulatta*) can influence on decision-making of human-macaque conflict mitigation? *Journal of Wildlife*, *3*(2), 52–62. https://doi.org/10.22120/jwb.2019.106831.1065

Ganguly, I., Chauhan, N. S., & Verma, P. (2018). Assessment of Human-Macaque Conflict and Possible Mitigation Strategies in and Around Asola-Bhatti Wildlife Sanctuary, Delhi NCR. *Journal of Environment & Ecology*, *36*(3), 823–827.

Ghosh, A., Thakur, M., Singh, S. K., Sharma, L. K., & Chandra, K. (2020). Gut microbiota suggests dependency of Arunachal Macaque (*Macaca munzala*) on anthropogenic food in Western Arunachal Pradesh, Northeastern India: Preliminary findings. *Global Ecology and Conservation*, *22*, e01030. https://doi.org/10.1016/j.gecco.2020.e01030

Gumert, M. D., Hamada, Y., & Malaivijitnond, S. (2013). Human activity negatively affects stone tool-using Burmese long-tailed macaques *Macaca fascicularis aurea* in Laem Son National Park, Thailand. *Oryx*, *47*(4), 535–543. https://doi.org/10.1017/S0030605312000130

Hardwick, J. L., Priston, N. E. C., Martin, T. E., Tosh, D. G., Mustari, A. H., & Abernethy, K. E. (2017). Community perceptions of the crop-feeding Buton macaque (*Macaca ochreata brunnescens*): an ethnoprimatological study on Buton Island, Sulawesi. *International Journal of Primatology*, *38*(6), 1102–1119. https://doi.org/10.1007/s10764-017-9999-0

Holzner, A., Ruppert, N., Swat, F., Schmidt, M., Weiß, B. M., Villa, G., Mansor, A., Mohd Sah, S. A., Engelhardt, A., Kühl, H., & Widdig, A. (2019). Macaques can contribute to greener practices in oil palm plantations when used as biological pest control. In *Current Biology* (Vol. 29, Issue 20, pp. R1066–R1067). Elsevier. https://doi.org/10.1016/j.cub.2019.09.011

Honda, T., Yamabata, N., Iijima, H., & Uchida, K. (2019). Sensitization to human decreases human-wildlife conflict: empirical and simulation study. *European Journal of Wildlife Research*, *65*(71). https://doi.org/10.1007/s10344-019-1309-z

Huang, C., Li, X. Y., Shi, L. J., & Jiang, X. L. (2018). Patterns of human-wildlife conflict and compensation practices around Daxueshan Nature Reserve, China. *Zoological Research*, *39*(6), 406–412. https://doi.org/10.24272/j.issn.2095-8137.2018.056

Ilham, K., Rizaldi, Nurdin, J., & Tsuji, Y. (2017). Status of urban populations of the long-tailed macaque (*Macaca fascicularis*) in West Sumatra, Indonesia. *Primates*, *58*(2), 295–305. https://doi.org/10.1007/s10329-016-0588-1

Ilham, K., Rizaldi, Nurdin, J., & Tsuji, Y. (2018). Effect of provisioning on the temporal variation in the activity budget of urban long-tailed macaques (*Macaca fascicularis*) in West Sumatra, Indonesia. *Folia Primatologica*, *89*(5), 347–356. https://doi.org/10.1159/000491790

Islam, A., Hossain, M. E., Haider, N., Rostal, M. K., Mukharjee, S. K., Ferdous, J., Miah, M., Rahman, M., Daszak, P., Rahman, M. Z., & Epstein, J. H. (2020). Molecular characterization of group A rotavirus from rhesus macaques (*Macaca mulatta*) at human–wildlife interfaces in Bangladesh. *Transboundary and Emerging Diseases*, *67*(2), 956–966. https://doi.org/10.1111/tbed.13431

Jeganathan, P., Mudappa, D., Raman, T. R. S., & Kumar, M. (2019). Understanding perceptions of people towards Lion-Tailed macaques in a fragmented landscape of the Anamalai Hills, Western Ghats, India. *Primate Conservation*, *32*(January), 11.

Ji, Y., Wei, X., Liu, F., Li, D., & Li, J. (2022). Spatial-temporal patterns of human-wildlife conflicts under coupled impact of natural and anthropogenic factors in Mt. Gaoligong, western Yunnan, China. *Global Ecology and Conservation*, *40*(August), e02329. https://doi.org/10.1016/j.gecco.2022.e02329

Jia, R., Wen, X., Guo, Y., Xiao, L., Feng, Y., & Li, N. (2022). Decline in cryptosporidium infection in free-ranging rhesus monkeys in a park after public health interventions. *Frontiers in Cellular and Infection Microbiology*, *12*(July), 1–7. https://doi.org/10.3389/fcimb.2022.901766

Kalam, T., Puttaveeraswamy, T. A., Srivastava, R. K., Puyravaud, J. P., & Davidar, P. (2020). Spatial aggregation and specificity of incidents with wildlife make tea plantations in southern India potential buffers with protected areas. *Journal of Threatened Taxa*, *12*(11), 16478–16493. https://doi.org/10.11609/jott.5590.12.11.16478-16493

Karanth, K. K., & Kudalkar, S. (2017). History, location, and species matter: Insights for human–wildlife conflict mitigation from India. *Human Dimensions of Wildlife*, *22*(4), 331–346. https://doi.org/10.1080/10871209.2017.1334106

Klegarth, A. R., Hollocher, H., Jones-Engel, L., Shaw, E., Lee, B. P. Y. H., Feeney, T., Holmes, D., Laguea, D., & Fuentes, A. (2017). Urban primate ranging patterns: GPS-collar deployments for *Macaca fascicularis* and *M. sylvanus*. *American Journal of Primatology*, *79*(5), 1–17. https://doi.org/10.1002/ajp.22633

Knight, J. (2017). Wildlife tourism as crop protection? Double-goal provisioning and the transvaluation of the macaque in postwar Japan. *Human–Wildlife Interactions*, *11*(2), 217–230.

Koirala, S., Baral, S., Garber, P. A., Basnet, H., Katuwal, H. B., Gurung, S., Rai, D., Gaire, R., Sharma, B., Pun, T., & Li, M. (2022). Identifying the environmental and anthropogenic causes, distribution, and intensity of human rhesus macaque conflict in Nepal. *Journal of Environmental Management*, *316*(May), 115276. https://doi.org/10.1016/j.jenvman.2022.115276

Koirala, S., Garber, P. A., Somasundaram, D., Katuwal, H. B., Ren, B., Huang, C., & Li, M. (2021). Factors affecting the crop raiding behavior of wild rhesus macaques in Nepal: Implications for wildlife management. *Journal of Environmental Management*, *297*(February), 113331. https://doi.org/10.1016/j.jenvman.2021.113331

Kyes, P., Thamsenanupap, P., Tanee, T., Intralawan, A., & Kyes, R. C. (2018). Previously unreported population of rhesus macaques *Macaca mulatta* in Chiang Rai province, Thailand: Preliminary observations. *Asian Primates Journal*, *7*(1), 6–13. http://www.ncbi.nlm.nih.gov/pubmed/31844847%0Ahttp://www.pubmedcentral.nih.gov/articlerender.fcgi?artid=PMC6914309

Lane-deGraaf, K. E., Kennedy, R. C., Arifin, S. M. N., Madey, G. R., Fuentes, A., & Hollocher, H. (2013). A test of agent-based models as a tool for predicting patterns of pathogen transmission in complex landscapes. *BMC Ecology*, *13*(35), 1–12. https://doi.org/10.1186/1472-6785-13-35

Lee, M. H., Rostal, M. K., Hughes, T., Sitam, F., Lee, C. Y., Japning, J., Harden, M. E., Griffiths, A., Basir, M., Wolfe, N. D., Epstein, J. H., & Daszak, P. (2015). Macacine herpesvirus 1 in long-tailed macaques, Malaysia, 2009-2011. *Emerging Infectious Diseases*, *21*(7), 1107–1113. https://doi.org/10.3201/eid2107.140162

Lee, W. N., & Davey, G. (2015). Chinese visitors’ experiences of nature and wild macaques: inspiration and personal growth for living in Hong Kong. *Human Dimensions of Wildlife*, *20*(3), 206–219. https://doi.org/10.1080/10871209.2015.1004468

Li, W., & von Essen, E. (2021). Guarding crops from monkey troops: farmer-monkey interaction near a nature reserve in Guangxi, China. *Environmental Sociology*, *7*(1), 12–24. https://doi.org/10.1080/23251042.2020.1811004

Maibeche, Y., Moali, A., Yahi, N., & Menard, N. (2015). Is diet flexibility an adaptive life trait for relictual and peri-urban populations of the endangered primate Macaca sylvanus? *PLoS ONE*, *10*(2), e0118596. https://doi.org/10.1371/journal.pone.0118596

Md-Zain, B. M., Ruslin, F., & Idris, W. M. R. (2014). Human-macaque conflict at the main campus of Universiti Kebangsaan Malaysia. *Pertanika Journal of Tropical Agricultural Science*, *37*(1), 73–85.

Milda, D., Ramesh, T., Kalle, R., Gayathri, V., & Thanikodi, M. (2020). Ranger survey reveals conservation issues across Protected and outside Protected Areas in southern India. *Global Ecology and Conservation*, *24*, e01256. https://doi.org/10.1016/j.gecco.2020.e01256

Mochizuki, S., & Murakami, T. (2013). Scale dependent effects in resource selection by crop-raiding Japanese macaques in Niigata Prefecture, Japan. *Applied Geography*, *42*, 13–22. https://doi.org/10.1016/j.apgeog.2013.04.016

Nahallage, C. A. D., Dasanayake, D. A. M., Hewamanna, D. T., & Ananda, D. T. H. (2022). Utilization of home garden crops by primates and current status of human-primate interface at Galigamuwa Divisional Secretariat Division in Kegalle District, Sri Lanka. *Journal of Threatened Taxa*, *14*(1), 20478–20487. https://doi.org/10.11609/jott.7560.14.1.20478-20487

Nandi, J. S., Rathore, S. S., & Mathur, B. R. (2021). Transmission of infectious viruses in the natural setting at human-animal interface. *Current Research in Virological Science*, *2*(March), 100008. https://doi.org/10.1016/j.crviro.2021.100008

Neha, S. A., Hasan, M. A. U., Baki, M. A., & Sehrin, S. (2021). Rhesus macaque *Macaca mulatta* (Mammalia: Primates: Cercopithecidae) in a human-modified landscape: population, activity budget, and societal perceptions in Bangladesh. *Journal of Threatened Taxa*, *13*(9), 19203–19211. https://doi.org/10.11609/jott.7073.13.9.19203-19211

Nekaris, K. A. I., Boulton, A., & Nijman, V. (2013). An ethnoprimatological approach to assessing levels of tolerance between human and commensal non-human primates in Sri Lanka. *Journal of Anthropological Sciences*, *91*, 219–231. https://doi.org/10.4436/JASS.91008

Nijman, V., Spaan, D., Rode-Margono, E. J., Wirdateti, & Nekaris, K. A. I. (2017). Changes in the primate trade in indonesian wildlife markets over a 25-year period: Fewer apes and langurs, more macaques, and slow lorises. *American Journal of Primatology*, *79*(11), 1–13. https://doi.org/10.1002/ajp.22517

Pal, A., Mahato, S., Leca, J. B., & Sinha, A. (2022). Blowing the lid off! Bottle-directed, extractive foraging strategies in synurbic bonnet macaques *Macaca radiata* in southern India. *Frontiers in Psychology*, *13*(December), 1–18. https://doi.org/10.3389/fpsyg.2022.973566

Paudel, P. K. (2017). Conflict due to Assamese Macaques (*Macaca assamensis* McClelland 1840) and crop protection strategies in Kali-gandaki River Basin, Western Nepal. *Our Nature*, *14*(1), 107–114. https://doi.org/10.3126/on.v14i1.16449

Pebsworth, P., & Radhakrishna, S. (2020). Using conditioned taste aversion to reduce human-nonhuman primate conflict: A comparison of four potentially illness-inducing drugs. *Applied Animal Behaviour Science*, *225*, 104948. https://doi.org/10.1016/j.applanim.2020.104948

Pilot, M., & Majolo, B. (2020). *Effect of human activity on habitat selection in the endangered Barbary macaque*. *23*, 373–385. https://doi.org/10.1111/acv.12543

Poornima, A. M. N. S., Weerasekara, W. M. L. S., Vinobaba, M., & Karunarathna, K. A. N. K. (2022). Community-level awareness and attitudes towards human–monkey conflict in Polonnaruwa district, Sri Lanka. *Primates*, *63*(3), 261–270. https://doi.org/10.1007/s10329-022-00985-3

Qiao, C. W., & Ruppert, N. (2017). Preliminary assessment of distribution and behaviours of primates at Penang National Park, Malaysia. *Malayan Nature Journal*, *69*(February), 345–351.

Radford, L., Alexander, S., & Waters, S. (2018). On the rocks: Using discourse analysis to examine relationships between Barbary macaques (*Macaca sylvanus*) and people on Gibraltar. *Folia Primatologica*, *89*(1), 30–44. https://doi.org/10.1159/000485125

Regmi, G. R., Nekaris, K. A. I., Kandel, K., & Nijman, V. (2013). Crop-raiding macaques: Predictions, patterns and perceptions from Langtang National Park, Nepal. *Endangered Species Research*, *20*(3), 217–226. https://doi.org/10.3354/esr00502

Riley, E. P., Shaffer, C. A., Trinidad, J. S., Morrow, K. S., Sagnotti, C., Carosi, M., & Ngakan, P. O. (2021). Roadside monkeys: anthropogenic effects on moor macaque (*Macaca maura*) ranging behavior in Bantimurung Bulusaraung National Park, Sulawesi, Indonesia. *Primates*, *62*(3), 477–489. https://doi.org/10.1007/s10329-021-00899-6

Riley, E. P., Tolbert, B., & Farida, W. R. (2013). Nutritional content explains the attractiveness of cacao to crop raiding Tonkean macaques. *Current Zoology*, *59*(2), 160–169. https://doi.org/10.1093/czoolo/59.2.160

Riley, E. P., & Wade, T. W. (2016). Adapting to florida’s riverine woodlands: The population status and feeding ecology of the silver river rhesus macaques and their interface with humans. *Primates*, *57*(2), 195–210. https://doi.org/10.1007/s10329-016-0517-3

Saito, M. U., Momose, H., Inoue, S., Kurashima, O., & Matsuda, H. (2016). Range-expanding wildlife: modelling the distribution of large mammals in Japan, with management implications. *International Journal of Geographical Information Science*, *30*(1), 20–35. https://doi.org/10.1080/13658816.2014.952301

Saraswat, R., Sinha, A., & Radhakrishna, S. (2015). A god becomes a pest? Human-rhesus macaque interactions in Himachal Pradesh, northern India. *European Journal of Wildlife Research*, *61*(3), 435–443. https://doi.org/10.1007/s10344-015-0913-9

Schurer, J. M., Ramirez, V., Kyes, P., Tanee, T., Patarapadungkit, N., Thamsenanupap, P., Trufan, S., Grant, E. T., Garland-Lewis, G., Kelley, S., Nueaitong, H., Kyes, R. C., & Rabinowitz, P. (2019). Long-tailed macaques (*Macaca fascicularis*) in urban landscapes: Gastrointestinal parasitism and barriers for healthy coexistence in northeast Thailand. In *American Journal of Tropical Medicine and Hygiene* (Vol. 100, Issue 2, pp. 357–364). https://doi.org/10.4269/ajtmh.18-0241

Sengupta, A., McConkey, K. R., & Radhakrishna, S. (2015). Primates, provisioning and plants: Impacts of human cultural behaviours on primate ecological functions. *PLoS ONE*, *10*(11), 1–13. https://doi.org/10.1371/journal.pone.0140961

Sengupta, A., & Radhakrishna, S. (2020). Factors predicting provisioning of macaques by humans at tourist sites. *International Journal of Primatology*, *41*, 471–485. https://doi.org/10.1007/s10764-020-00148-5

Sengupta, A., Widayati, K. A., Tsuji, Y., Yanti, R., Rahman, M. F., Balakrishna, N., & Radhakrishna, S. (2021). Why do people visit primate tourism sites? Investigating macaque tourism in Japan and Indonesia. *Primates*, *62*(6), 981–993. https://doi.org/10.1007/s10329-021-00951-5

Sha, J. C. M., & Hanya, G. (2013a). Diet, activity, habitat use, and ranging of two neighboring groups of food-enhanced long-tailed macaques (*Macaca fascicularis*). *American Journal of Primatology*, *75*(6), 581–592. https://doi.org/10.1002/ajp.22137

Sha, J. C. M., & Hanya, G. (2013b). Temporal food resource correlates to the behavior and ecology of food-enhanced long-tailed macaques (*Macaca fascicularis*). *Mammal Study*, *38*(3), 163–175. https://doi.org/10.3106/041.038.0305

Shano, S., Islam, A., Hagan, E., Rostal, M. K., Martinez, S., Al Shakil, A., Hasan, M., Francisco, L., Husain, M. M., Rahman, M., Flora, M. S., Miller, M., Daszak, P., & Epstein, J. H. (2021). Environmental Change and Zoonotic Disease Risk at Human-Macaque Interfaces in Bangladesh. *EcoHealth*, *18*(4), 487–499. https://doi.org/10.1007/s10393-021-01565-5

Supriatna, J., Shekelle, M., Fuad, H. A. H. H., Winarni, N. L., Dwiyahreni, A. A., Farid, M., Mariati, S., Margules, C., Prakoso, B., & Zakaria, Z. (2020). Deforestation on the Indonesian island of Sulawesi and the loss of primate habitat. *Global Ecology and Conservation*, *24*, e01205. https://doi.org/10.1016/j.gecco.2020.e01205

Teampanpong, J. (2021). Improper garbage management attracts vertebrates in a Thai national park. *Ecoscience*, *28*(2), 107–113. https://doi.org/10.1080/11956860.2021.1872264

Trupti D et al; (2018). Dietary preference and feeding patterns of the urban rhesus macaque *Macaca mulatta* (Mammalia: Primates: Cercopithecidae) in Asola-Bhatti Wildlife Sanctuary in India. *Journal of Threatened Taxa*, *10*(January), 11391–11398.

Ueda, Y., Kiyono, M., Nagano, T., Mochizuki, S., & Murakami, T. (2018). Damage control strategies affecting crop-raiding Japanese macaque behaviors in a farming community. *Human Ecology*, *46*(2), 259–268. https://doi.org/10.1007/s10745-018-9994-x

Usui, R., Sheeran, L. K., Li, J. H., Sun, L., Wang, X., Pritchard, A. J., Duvall-Lash, A. S., & Steve Wagner, R. (2014). Park rangers’ behaviors and their effects on tourists and tibetan macaques (*Macaca thibetana*) at Mt. Huangshan, China. *Animals*, *4*(3), 546–561. https://doi.org/10.3390/ani4030546

Waters, S., Bell, S., & Setchell, J. M. (2018). Understanding human-animal relations in the context of primate conservation: A multispecies ethnographic approach in North Morocco. *Folia Primatologica*, *89*(1), 13–29. https://doi.org/10.1159/000480079

Waters, S., El Harrad, A., Bell, S., & Setchell, J. M. (2019). Interpreting people’s behavior toward primates using qualitative aata: a case study from North Morocco. *International Journal of Primatology*, *40*, 316–330. https://doi.org/10.1007/s10764-019-00087-w

Wenz-mücke, A., Sithithaworn, P., Petney, T. N., & Taraschewski, H. (2013). Human contact in fluences the foraging behaviour and parasite community in long-tailed macaques. *Parasitology*, 1–10. https://doi.org/10.1017/S003118201200203X

Young, Z., Jin-hua, L., Dong-po, X., Bing-hua, S., Yu-rui, X., Wang, X., & Dao, Z. (2013). Potential pathogen transmission risk in non-human primate ecotourism: A case study at Mt. Huangshan, China. *Life Science Journal*, *5*(1), 137–138. https://doi.org/10.21608/egjec.2013.94997

Zak, A. A., & Riley, E. P. (2017). Comparing the use of camera traps and farmer reports to study crop feeding behavior of Moor macaques (*Macaca maura*). *International Journal of Primatology*, *38*(2), 224–242. https://doi.org/10.1007/s10764-016-9945-6

Zamri, M. A., & Md-Zain, B. M. (2022). Long-tailed macaques of the Batu caves, Peninsular Malaysia: Population estimate, nuisance behaviors, and human perception. *Journal of Animal and Plant Sciences*, *32*(4), 1150–1158. https://doi.org/10.36899/JAPS.2022.4.0520
