# Supplementary material for: Ecosystem health appears neglected in the management of the human-macaque interface: A systematic review
Source: One Health. 2024 Aug 20;19:100875. doi: 10.1016/j.onehlt.2024.100875 (PMC11381846; doi:10.1016/j.onehlt.2024.100875)
Supplement: Supplementary file 2 — Supplementary material 2 [file mmc2.docx]

**Supplement 2.**


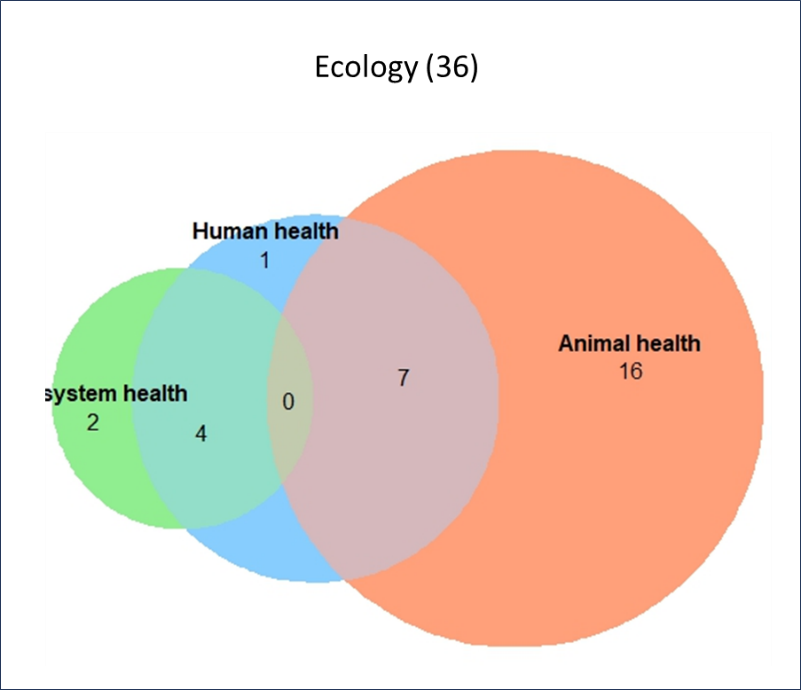


**Figure 1.** The attribution of publications (n=36) to the OH dimensions (human, blue; animal orange, ecosystem green) for HIM management activities of the publications that contained only ecology pillar.


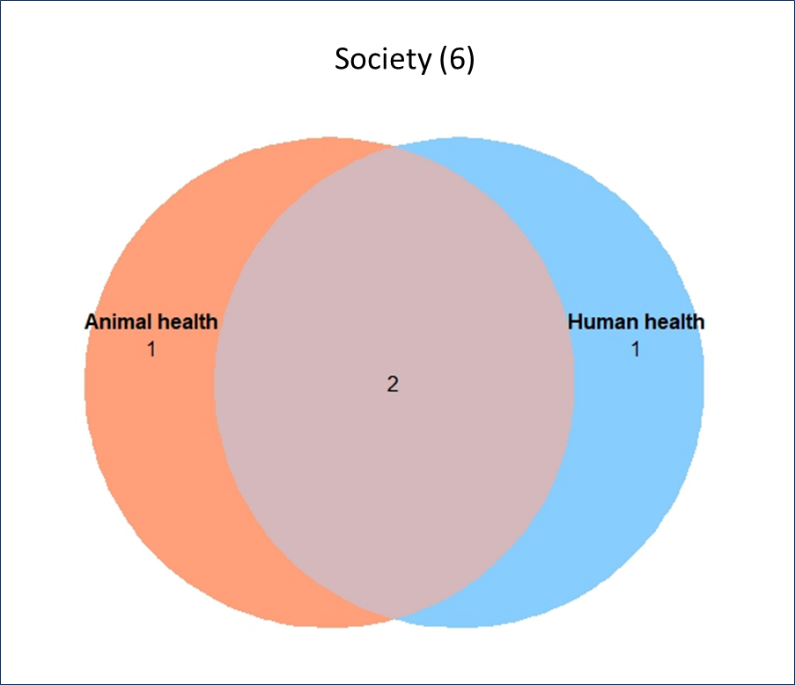


**Figure 2.** The attribution of publications (n=6) to the OH dimensions (human, blue; animal orange, ecosystem green) for HMI management activities of the publications that contained only society pillar.


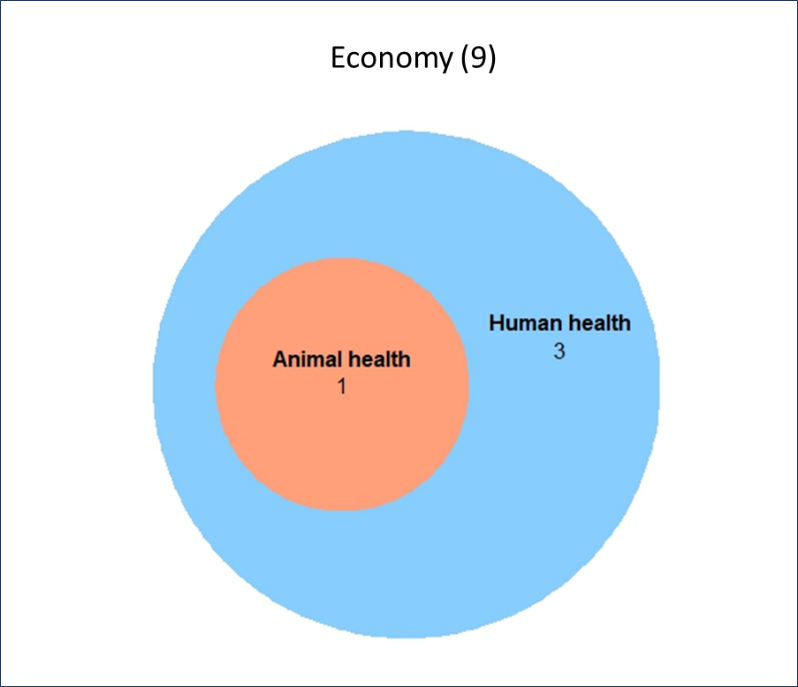


**Figure 3.** The attribution of publications (n=9) to the OH dimensions (human, blue; animal orange, ecosystem green) for HMI management activities of the publications that contained only economy pillar.


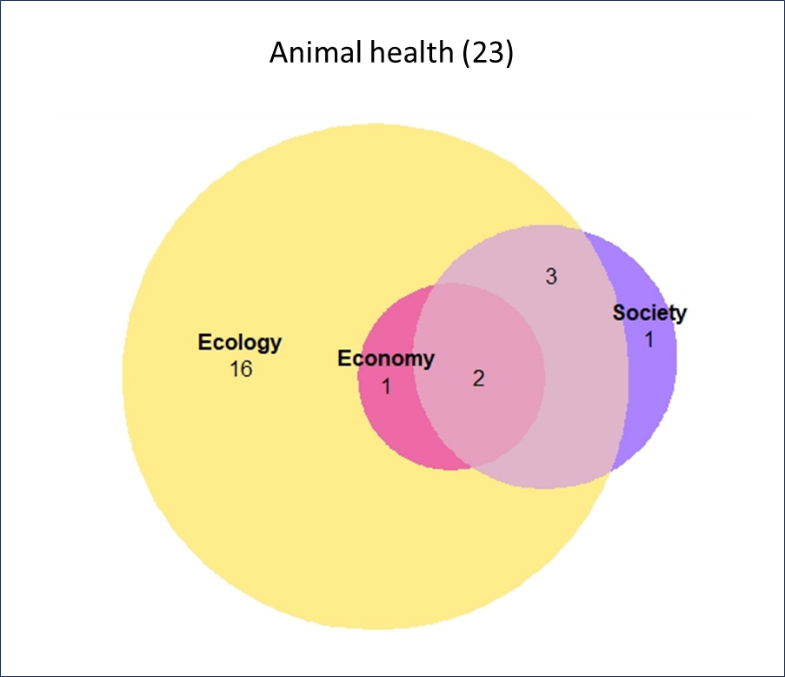


**Figure 4.** The attribution of publications (n=23) to the pillars of sustainability (ecology yellow, society purple, economy pink) of the publications that contained only animal health domain for HMI management activities.


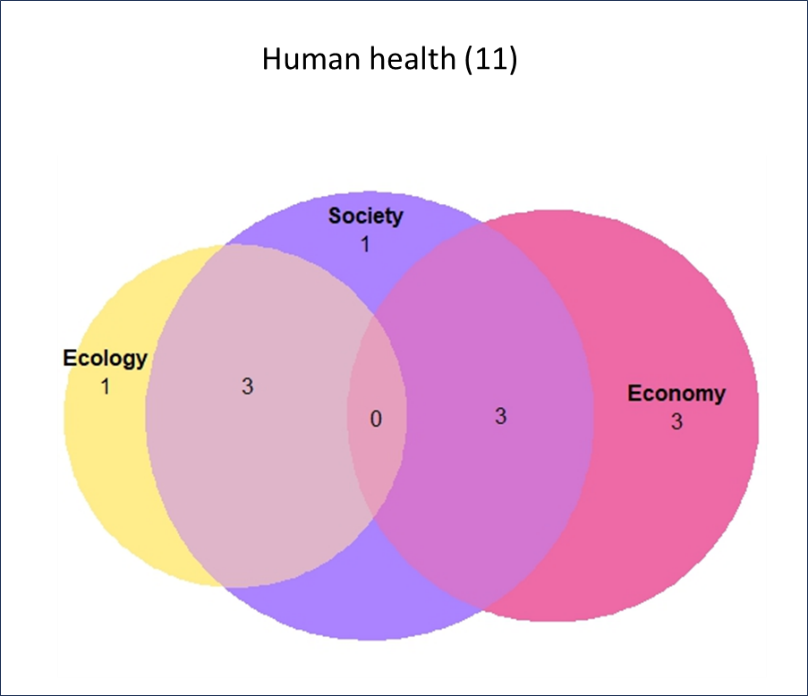


**Figure 5.** The attribution of publications (n=11) to the pillars of sustainability (ecology yellow, society purple, economy pink) of the publications that contained only human health domain for HMI management activities.


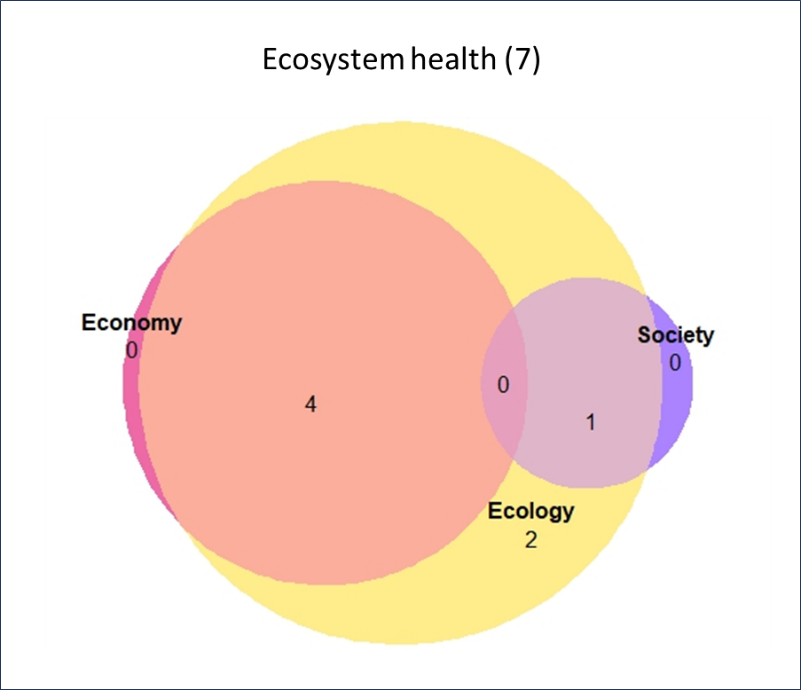


**Figure 6.** The attribution of publications (n=7) to the pillars of sustainability (ecology yellow, society purple, economy pink) of the publications that contained only ecosystem health domain for HMI management activities.
